# Oral [^18^F]-Fluoro-Thia-Heptadecanoic Acid Positron Emission Tomography Reveals Mesenteric-to-Central Lymphatic Flow

**DOI:** 10.1016/j.gastha.2026.100956

**Published:** 2026-04-08

**Authors:** Daniel D. Lee, Richard Laforest, Alexander Ushinsky, Michael L. Nickels, Robert J. Gropler, Gwendalyn J. Randolph, the FTHA Study Working Group, Heyun Lee, Kitty Harrison, Nicholas Dunn, Christopher G. Huckstep, Quazim Alayo, Bernd H. Zinseleyer, Shelei Pan, Adam Neff, Ben Stawicki

**Affiliations:** 1 Department of Pathology and Immunology, Washington University School of Medicine, St. Louis, Missouri; 2 Department of Radiology, Mallinckrodt Institute of Radiology, Washington University School of Medicine, St. Louis, Missouri

Interstitial fluid continuously arises in tissues and returns to plasma through the one-way flow system of the lymphatic vasculature. The body's central lymphatic vessel returns lymph to the venous circulation.^1^^,^^2^ Lymphatic leaks or pathologic collaterals can arise from congenital or acquired pathologies like chylothorax or protein-losing enteropathy.^1^^,^^2^ For management of these conditions, central lymphatic imaging is essential. Current state-of-the-art imaging relies on dynamic contrast-enhanced magnetic resonance lymphangiography.^1^^,^^3^^,^^4^ Due to its invasive nature, this procedure is rarely employed in healthy individuals.^4^ Dynamic contrast-enhanced magnetic resonance lymphangiography of abdominal organs such as the liver or the mesentery is technically challenging and limited to a few referral centers. The majority of what is known about central lymphatic outflow in healthy subjects arises from cadaveric studies.^1^^,^^5^^,^^6^ Here, following informed consent in a protocol approved by Washington University’s Institutional Review Board (protocol #202403135) and the Radioactive Drug Research Committee (protocol #981F), we evaluated the fatty acid radiotracer [^18^F]-fluoro-thia-heptadecanoic acid ([^18^F]-FTHA) as a positron emission tomography (PET) agent for imaging of the mesenteric and central lymphatic vasculature. When given orally, [^18^F]-FTHA incorporates into chylomicrons^7^^,^^8^ that enter the lymph of the small bowel and drain through the mesenteric lymphatics to the TD to deliver fatty nutrients to the body.^2^

We combined a 1.4 mCi (range, 1.2–1.5 mCi) dose of [^18^F]-FTHA into a 236 mL liquid mixed meal based on the nutritional drink BOOST that was consumed by each participant over a 10-minute interval, followed by ingestion of 118 mL of the same drink without [^18^F]-FTHA and 118 mL of water. Serial PET/computed tomography (CT) imaging was initiated 20 minutes after the drink was started. Radiation dosimetry and biodistribution estimates were obtained in 7 healthy individuals (3 males, 4 females) by sequential whole-body continuous bed motion PET/CT acquisition from vertex to upper thigh starting from completion of the drink and repeated at 2, 4, and 6 hours to establish tracer biodistribution. Time-integrated activity coefficients (TIACs) were calculated (Supplementary Figure D) and organ-specific activity determined (Supplementary Table). TIACs for different organs from a representative individual are shown in Supplementary Figure E–H.

Tracer activity appeared immediately in the stomach and duodenum, with low contrast activity also retained in the mouth and esophagus (Figure A). Within 20 minutes after imaging began, faint activity that increased over time was apparent in the TD and at the LVJ (Figure A, blue arrows) and in mesenteric regions between small intestinal bowel loops (Figure A, red outlines). [^18^F]-FTHA in the TD was readily segmented from the esophagus (Figure B), including in individuals with 2 TD termini (Figure C).

**Figure:**
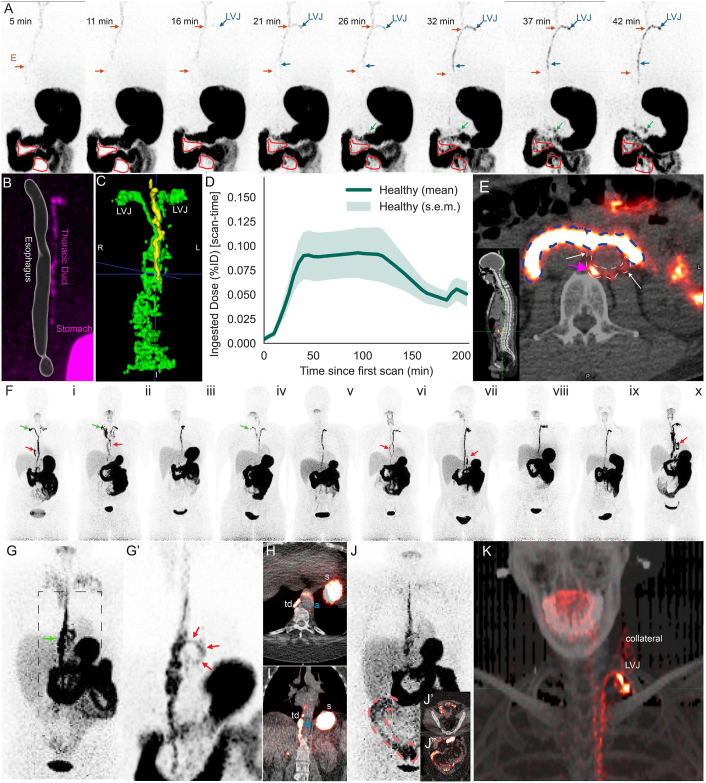
Oral [^18^F]-FTHA PET/CT visualizes mesenteric-to-central lymphatic transport. (A) Serial, coronal frames of maximum intensity projection (MIP) PET signal after oral [^18^F]-FTHA. Esophagus (E; orange arrows) and TD (blue arrows), including the LVJ of the TD near the clavicle, are annotated. Regions of mesentery between bowel loops are outlined in red. (B) TotalSegmentator-derived segmentation of esophagus (light gray) overlaid on the PET signal (magenta) demonstrates TD near esophagus. (C) Representative 3D surface rendering after voxel-wise segmentation of lymphatic tracer signal (green) vs esophageal signal (yellow) in healthy subject. (D) LVJ time–activity curve in healthy participants (percent ingested dose, %ID, over time since first scan; arithmetic mean ± SEM, *n* = 10). (E) Axial PET/CT overlay at the level of mid abdomen demonstrating the PET signal within the small intestine lumen (blue dashes), with lymphatic vessels bearing [^18^F]-FTHA (white arrows, white dotted lines) extending from the intestine to the TD (magenta arrow). (F) Whole-body MIPs from each healthy participant (numbered i-x) showing PET signal in the TD outflow terminating at 1 or 2 LVJs. Green arrows indicate 2 TD termini (dual LVJ) variants; red arrows indicate prominent secondary lymphatic trunk outflow alongside the TD. (G) At approximately 100 minutes, MIP PET in a participant with lifelong PLE showing an engorged TD segment (green arrow) without signal reaching a distinct LVJ near the clavicle; boxed region indicates the area magnified in (G’). (G’) Magnified MIP highlighting abnormal central lymphatic anatomy along with lymphatic collateral channel (red arrows). (H and I) Axial (H) or coronal (I) PET/CT overlay in PLE patient demonstrating abnormal [^18^F]-FTHA-containing pathway looping around the esophagus and descending aorta (a, blue dashed); stomach (s). (J) Delayed MIP at approximately 220 minutes in the same PLE patient showing prominent colonic [^18^F]-FTHA tracer activity (red dashed outline), with PET/CT confirmation on (J’) axial and (J”) coronal views. (K) MIP PET/CT in a primary lymphedema patient demonstrating TD and LVJ signal, with collateral signal further extending aberrantly into the neck. PLE, protein-losing enteropathy.

Activity extraluminal to the gastrointestinal tract was surmised to correspond with [^18^F]-FTHA secretion of chylomicrons into the mesenteric and central lymphatic outflow (TD). [^18^F]-FTHA diluted abruptly upon entering faster flowing blood, represented by a sharp drop in PET signal at the region of the LVJ (Figure A, Supplementary Figure A). [^18^F]-FTHA activity within the TD and mesenteric lymphatics of the small intestine appeared simultaneously (Figure A). At early time points, signal in organs like the heart and liver was low (Supplementary Figure B), but following delivery of [^18^F]-FTHA to the systemic circulation where lipoprotein lipase releases fatty acids from chylomicrons that reach the blood,^7^ [^18^F]-FTHA activity increased in these organs while remaining lower than signal in the upper gastrointestinal and TD (Supplementary Figure C). The highest organ dose and longest TIACs were observed at the stomach wall with a sex-averaged dose of 1.36 rad/mCi (Supplementary Table). The sex-averaged effective dose (103) was 0.22 rem/mCi (59.5 μSv/MBq) or 3.0 mSv per 50 MBq (Supplementary Table).

To approximate lymph flow rate, we segmented several centimeters of the terminal end of the TD starting from where it angled away from the esophagus to the LVJ (Supplementary Figure I). The initial delivery rate of [^18^F]-FTHA across the LVJ rose at 0.00511% ± 0.00128% ingested dose (ID)/min, reaching ∼0.0929% ± 0.0253% ID at 53.6 ± 9.48 minutes before declining by ∼110 minutes (Figure D). Area under the curve analysis indicated that 13.7 ± 3.4% ID passed through the LVJ during the 220-minute period investigated. Distinct paths of PET signal were observed between the intestine and the TD on PET/CT, representing mesenteric lymphatic vessels (Figure E, white arrows and white dotted line).

Inclusive of our dosimetry analysis, we investigated [^18^F]-FTHA outflow through central lymphatics in 10 healthy individuals (6 females, 4 males; age 28–62 years; BMI 20–32 kg/m^2^) who had not been diagnosed with suspected lymphatic disorders. Three of the 10 healthy subjects had two TD termini (Figure F, green arrows), an uncommon but known variation in the central lymphatic system.^9^ We also observed prominent contrast in a secondary trunk near the TD in at least half of the healthy participants (Figure F, red arrows), along with additional lymphatic collaterals in some individuals (Figure F, panel x). These secondary trunks were apparent after surface rendering to remove stomach and intestinal signal (Figure C). Segmentation of the esophagus confirmed that the signal within the collateral trunk was distinct from the esophagus (Figure C). These secondary excursions are not well described in existing literature and suggest that central lymphatic pathways are not yet fully defined in healthy humans. It is possible that lymph trunks thought to feed into the TD that lack valves^10^ receive reflux from the TD in some settings, such as in the postprandial state studied here.

We next recruited a patient with lifelong protein-losing enteropathy (Figure G–J). When given oral [^18^F]-FTHA in our protocol, findings in this patient suggested possible central lymphatic flow obstruction with secondary flow via lymphatic collaterals. [^18^F]-FTHA activity was present in a highly dilated segment of the TD (Figure G, green arrow; magnified in Figure G’) without activity apparent at the LVJ. Vague signal in the pulmonary system was present (Figure G), possibly representing retrograde flow into pulmonary lymphatics. At the site of TD dilation, activity adjacent to the esophagus and descending aorta was identified (Figure H and I, aorta outlined in blue), which could represent collateral flow. By 220 minutes, a signal was observed in the colon (Figure J and J”), an unusual outcome for dietary fatty acid in normal subjects, but observed in one of the healthy participants (Figure F, panel x). [^18^F]-FTHA signal (dietary fat) in the colon may be consistent with lymphatic pathologies driving protein-losing enteropathy and account for the patient’s propensity to have >5 bowel movements per day.

Finally, a primary lymphedema patient transported lymph to the LVJ at the subclavian vein, but collaterals carrying [^18^F]-FTHA extended from the LVJ into the neck (Figure K), which may reflect backflow into cervical lymphatic channels due to partial LVJ obstruction.

We conclude that oral [^18^F]-FTHA–PET/CT facilitates safe and effective noninvasive, dynamic imaging of mesenteric-to-central lymphatic transport and has potential to illuminate a better understanding of mesenteric-to-central lymph flow in health and disease.

## Figures and Tables

**Supplementary Figure:**
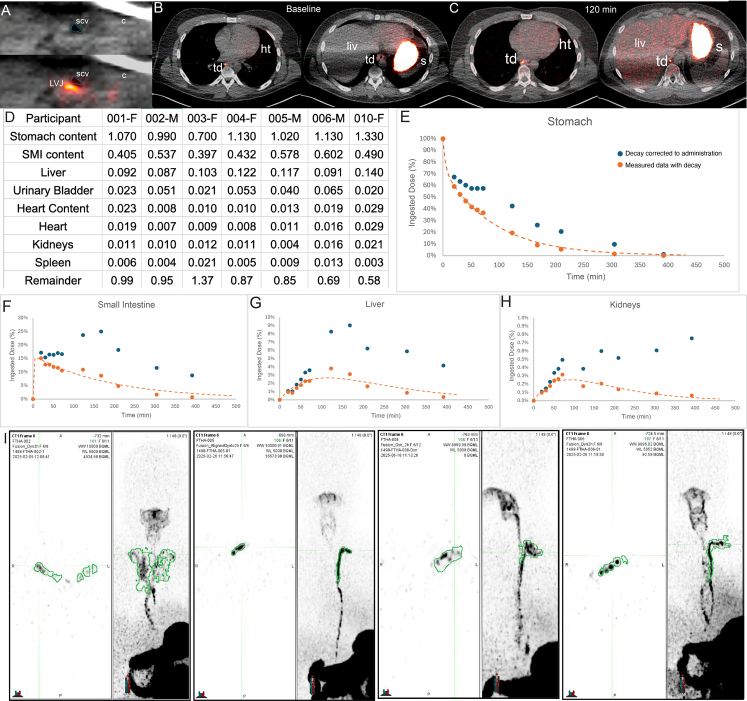
Quantification workflow and dosimetry time–activity data for oral [<sup>^18^F]-FTHA PET/CT. (A) Axial PET/CT example identifying the LVJ relative to the subclavian vein (scv) and clavicle (c), with focal PET signal at the LVJ. (B and C) Representative axial PET/CT overlays at initial baseline scanning (panel B) or at 120 minutes (panel C) showing TD signal (td) relative to heart (ht) or liver (liv). (D) Organ time-integrated activity coefficients (TIACs; in hours) tabulated for the 7 dosimetry participants, including stomach contents, small intestine (SMI) contents, liver, urinary bladder, cardiac blood pool/wall, kidneys, spleen, and remainder. (E–H) Representative time–activity curves (percent ingested dose vs time) for stomach (E), small intestine contents (F), liver (G), and kidneys (H), shown as both decay-corrected to administration and scan-time measured activity (with physical decay applied). (I) MIM software-based quantification workflow illustrating voxel of interest placement and segmentation for TD/LVJ analysis (green) for 4 individuals used to generate data in Figure D, with axial PET views on the left for each individual and the corresponding coronal MIP rendering on right. MIP, maximum intensity projection.

## Supplementary Materials

Supplementary Table

Extended PDF
