# Supplementary material for: Oral [18F]-Fluoro-Thia-Heptadecanoic Acid Positron Emission Tomography Reveals Mesenteric-to-Central Lymphatic Flow
Source: Gastro Hep Adv. 2026 Apr 8;5(6):100956. doi: 10.1016/j.gastha.2026.100956 (PMC13186019; doi:10.1016/j.gastha.2026.100956)
Supplement: Supplementary Material [file mmc1.pdf]

Table S1. Human radiation dose for 18F-FTHA in humans. Doses can be converted to SI units by dividing by 3.7.

Organ dose (rad/mCi)	1-F	3-F	4-F	010-F	Female		2-M	5-M	6-M	Male		Sex Averaged	
Organ					Average +/- SD					Average +/- SD		Average +/- SD	
Adipose tissue	0.034	0.038	0.033	0.029	0.033	0.004	0.037	0.036	0.036	0.036	0.001	0.033	0.003
Adrenals	0.145	0.122	0.152	0.171	0.147	0.020	0.101	0.105	0.111	0.106	0.005	0.147	0.027
Bone - endosteal cells	0.033	0.038	0.031	0.028	0.032	0.004	0.026	0.025	0.023	0.025	0.002	0.032	0.005
Bone red marrow	0.053	0.056	0.052	0.051	0.053	0.002	0.043	0.042	0.041	0.042	0.001	0.053	0.006
Brain	0.020	0.027	0.018	0.012	0.019	0.006	0.016	0.015	0.012	0.014	0.002	0.019	0.005
Breast tissue	0.040	0.040	0.039	0.040	0.040	0.001	0.000	0.000	0.000	0.000	0.000	0.040	0.021
Colon - left	0.067	0.069	0.068	0.068	0.068	0.001	0.149	0.154	0.163	0.155	0.007	0.068	0.047
Colon - rectosigmoid	0.055	0.063	0.058	0.051	0.057	0.005	0.035	0.033	0.034	0.034	0.001	0.057	0.013
Colon - right	0.054	0.058	0.054	0.053	0.055	0.002	0.065	0.067	0.067	0.066	0.001	0.055	0.006
Esophagus	0.069	0.062	0.067	0.074	0.068	0.005	0.065	0.067	0.070	0.067	0.002	0.068	0.004
Eye lens	0.016	0.021	0.014	0.010	0.015	0.004	0.012	0.011	0.009	0.011	0.002	0.015	0.004
Gallbladder wall	0.129	0.114	0.138	0.154	0.134	0.017	0.088	0.094	0.093	0.092	0.003	0.134	0.025
Heart wall	0.128	0.085	0.105	0.165	0.121	0.034	0.107	0.118	0.135	0.120	0.014	0.121	0.025
Kidneys	0.139	0.122	0.147	0.170	0.145	0.020	0.095	0.097	0.115	0.103	0.011	0.145	0.027
Liver	0.131	0.114	0.144	0.158	0.137	0.019	0.093	0.116	0.117	0.109	0.013	0.137	0.022
Lung - ICRP133	0.057	0.054	0.055	0.059	0.056	0.002	0.050	0.051	0.051	0.051	0.001	0.056	0.003
Muscle	0.033	0.039	0.032	0.028	0.033	0.004	0.026	0.025	0.023	0.025	0.001	0.033	0.005
Oral mucosa	0.031	0.041	0.027	0.020	0.030	0.009	0.025	0.023	0.019	0.022	0.003	0.030	0.007
Ovaries	0.056	0.065	0.059	0.051	0.058	0.006	0.000	0.000	0.000	0.000	0.000	0.058	0.031
Pancreas	0.223	0.184	0.234	0.264	0.226	0.033	0.185	0.193	0.202	0.194	0.009	0.226	0.030
Pituitary gland	0.022	0.030	0.020	0.014	0.022	0.007	0.017	0.016	0.013	0.015	0.002	0.022	0.006
Prostate	0.000	0.000	0.000	0.000	0.000	0.000	0.036	0.032	0.034	0.034	0.002	0.000	0.018
Salivary glands	0.022	0.029	0.020	0.014	0.021	0.006	0.017	0.015	0.013	0.015	0.002	0.021	0.006
Skin	0.024	0.028	0.023	0.020	0.024	0.003	0.019	0.019	0.017	0.018	0.001	0.024	0.004
Small intestine	0.192	0.184	0.202	0.221	0.200	0.016	0.196	0.206	0.214	0.205	0.009	0.200	0.013
Spleen	0.234	0.202	0.242	0.269	0.237	0.028	0.124	0.168	0.164	0.152	0.024	0.237	0.051
Stomach Wall	1.388	0.936	1.465	1.717	1.376	0.325	1.262	1.302	1.436	1.333	0.091	1.376	0.237
Testes	0.000	0.000	0.000	0.000	0.000	0.000	0.019	0.017	0.014	0.017	0.002	0.000	0.009
Thymus	0.035	0.040	0.031	0.030	0.034	0.004	0.029	0.029	0.027	0.028	0.001	0.034	0.004
Thyroid	0.027	0.034	0.025	0.020	0.027	0.006	0.023	0.022	0.020	0.022	0.002	0.027	0.005
Urinary bladder wall	0.061	0.068	0.080	0.055	0.066	0.011	0.060	0.052	0.065	0.059	0.007	0.066	0.009
Uterus	0.062	0.070	0.068	0.057	0.064	0.006	0.000	0.000	0.000	0.000	0.000	0.064	0.035
Whole body target	0.040	0.043	0.038	0.037	0.040	0.003	0.034	0.033	0.032	0.033	0.001	0.040	0.004
Effective Dose (rem/mCi)	0.221	0.168	0.232	0.283	0.226	0.048	0.202	0.208	0.225	0.212	0.012	0.220	0.035
