# Supplementary material for: Oral [18F]-Fluoro-Thia-Heptadecanoic Acid Positron Emission Tomography Reveals Mesenteric-to-Central Lymphatic Flow
Source: Gastro Hep Adv. 2026 Apr 8;5(6):100956. doi: 10.1016/j.gastha.2026.100956 (PMC13186019; doi:10.1016/j.gastha.2026.100956)
Supplement: Supplementary Table [file mmc2.pdf]

Supplemental Text - Methods

Participant characteristics and recruitment

This observational study recruited study participants ages 21–80. Approval was granted by Washington University institutional review board, protocol #202403135 and the Radioactive Drug Research Committee, protocol #981F. Written informed consent was obtained prior to enrollment. Healthy-subject inclusion criteria required no known history of, or clinical suspicion for, cardiac or lymphatic vascular disease. A second cohort included participants with lymphatic or related congenital anomalies. Exclusion criteria included pregnancy, body weight >300 lb, recreational drug use, current participation in other investigational or radiopharmaceutical studies, and inability to tolerate PET/CT (e.g., claustrophobia or inability to remain supine). Medications and supplements were reviewed and eligibility was determined case-by-case. Participants completed a brief health survey and focused physical examination prior to imaging. Twelve adult participants (10 healthy, 2 in lymphatic disorders cohort) were enrolled (8 females, 4 males). Adverse event monitoring included real-time symptom reporting, baseline and end-of-scan vital signs, structured symptom checklists, and pre-/post-scan electrocardiograms. Nausea, reflux, emesis, abdominal discomfort, and allergic symptoms were specifically queried. No adverse events were encountered.

Synthesis of FTHA

14-(R,S)-[^{18}F] Fluoro-6-thia-heptadecanoic acid (FTHA) was prepared at the Washington University Cyclotron Facility and Nuclear Pharmacy using a modification of a published method[1]. Briefly, Kryptofix-222 assisted radiofluorination of the tosylate precursor was followed by hydrolysis with KOH. Radiosynthesis was performed on a GE FX-2N module, and purified by semi-preparative HPLC and sterile filtered to provide a ready to use final product (total synthesis time ~80 minutes).

Imaging protocol and tracer administration

A 16-oz formulated liquid mixed meal validated in prior chylomicron tracer studies was prepared in the Washington University Clinical Research and Translational Unit metabolic kitchen (10.8 g Sol Carb, 413 g chocolate BOOST® Plus, 2.6 g canola oil, 0.116 g lecithin, 24 g water). In the Mallinckrodt Institute of Radiology Center for Clinical Imaging and Research (CCIR), approximately 1.4 mCi (range 1.2–1.5 mCi) of [^{18}F]-FTHA was added to an 8-oz portion in a sealed cup with radiation shielding; overnight-fasted participants consumed the tracer-containing portion over 10 minutes after an overnight fast, followed by 4 oz of the drink without tracer and then 4 oz of water. PET/CT was performed on a Siemens Biograph Vision-600. Imaging began ~10 minutes after completion of the drink (~20 minutes after drink initiation). The first hour was acquired as serial whole-body continuous-bed-motion (CBM) sweeps with frame durations of approximately 5–10 minutes per sweep (5–10 sweeps total, depending on protocol). Additional whole-body CBM acquisitions were obtained at ~2 hours (15 minutes) and at later timepoints (e.g., 4 hours and 6 hours; 30 minutes each). Participants exited the scanner between sessions and were repositioned on return. For anatomic localization and attenuation correction, a low-dose CT was acquired at each imaging session (120 kVp, 38.4 mm total collimation, 17 mAs effective; CT reconstructed at 3-mm slice thickness per protocol).

Imaging analysis

The primary high-activity compartments prioritized for dosimetry quantification were stomach contents, small intestine contents, and liver; kidneys, spleen, urinary bladder, and heart were also evaluated. Liver, kidneys, spleen, and stomach were segmented using MOOSE3 AI software[2, 3]. Masses of liver, kidneys, and spleen, were estimated from segmented volumes assuming a tissue density of 1.05 g/cm³. A single volume of interest (VOI) encompassing the whole heart was used to sample cardiac activity (myocardium and blood pool were not separable on visual assessment). Stomach, urinary bladder, and small intestine activity were quantified as total activity within PET-derived VOI volumes.

Organ time-integrated activities (TIAs, formerly known as residence times, in hours) for each organ were computed from time–activity data expressed as percent injected dose per organ using trapezoidal numerical integration and fit with exponential functions to extrapolate beyond the last imaging time point. For stomach contents, the time–activity curve was additionally fitted to a bi-exponential model using Microsoft Excel Solver and analytically integrated to obtain the TIA. The calculated TIAs were entered into MIRDCalc (v1)[4-6] for F-18 and using the either the adult ICRP human male and female anthropomorphic models, as appropriate. For cardiac portioning, a fraction of heart wall residence time was assigned to the heart content as defined by the ratio of heart content to heart wall mass, where the heart content and mass were taken from ICRP-106[7]. All unaccounted activity in the organs was assigned to the reminder of the body. Organ radiation dose and Effective Dose (IRCP-103) were reported.

Esophagus and gastrointestinal tract were segmented using MOOSE3 AI software[2] or TotalSegmentator[8], while voxel-by-voxel segmentation and surface rendering was performed on ITK-Snap. For 3D visualization, segmentation label maps (NIfTI) and the corresponding CT and PET image volumes (converted from DICOM to image stacks) were imported into Imaris (v10.1.1) using the Imaris File Converter and rendered as overlaid volumetric channels to display anatomical context and segmented structures in three dimensions.

Image registration and calculations
Serial PET datasets (dynamic first-hour frames and subsequent CBM acquisitions) were aligned across timepoints to correct for inter-scan repositioning using CT for anatomical guidance (MIM v7.3.7). The first-hour plus ~2-hour aligned PET/CT dataset was used as the reference, and later frames were registered using rigid followed by deformable registration; transforms were applied to the PET data to enable time-consistent sampling. A 3D VOI encompassing the thoracic duct from the diaphragmatic hiatus to the LVJ was delineated on a timepoint with clear duct activity and deformably propagated to all frames. Propagated VOIs were reviewed and edited frame-by-frame to exclude adjacent non-duct activity and to confirm anatomical concordance on PET/CT overlay.

VOI activity at each timepoint was obtained from the VOI export (using mean activity concentration and VOI volume). Time was expressed as minutes from the first scan. Exported PET values were decay-corrected to administration and converted to scan-time activity using the physical decay of F-18 ($T_{1/2}$ = 109.77 min). Percent injected dose was calculated as $\%ID_{\mathrm{scan}} = 100 \times A_{\mathrm{VOI,scan}}/A_{\mathrm{inj}}$, with A_{inj} converted to Bq. When bilateral LVJ VOIs were available, LVJ_{Total} was computed per timepoint by summing left and right VOI activities (and volumes), and %ID were calculated from the combined VOI. For healthy-group summaries, individual %ID

time–activity curves were restricted to 0–240 min and linearly interpolated onto a uniform 5-min grid; interpolation was limited to each subject's observed time range (no extrapolation). At each grid timepoint, the group mean along with standard error of mean was calculated across available subjects. Because LVJ time–activity curves frequently exhibited a sustained plateau rather than a sharp peak, a "near-max time" was defined as the first grid time at which the group-mean curve reached ≥95% of its maximum value and remained above this threshold for ≥3 consecutive grid points (15 minutes). Group peak %ID was defined as the maximum of the group-mean curve. Delivery rate was defined as the time-derivative of %ID (%ID/min) using a rolling k-point linear regression (k=3) by fitting $\%ID = mt + b$ within each window and taking the maximum positive slope m as the peak rolling delivery rate, reported at the window's median time. Peak delivery time was estimated at the window's maximum.

References

1. Savisto N, Viljanen T, Kokkomaki E, et al. Automated production of [(18) F]FTHA according to GMP. J Labelled Comp Radiopharm 2018;61:84-93.
2. Shiyam Sundar LK, Yu J, Muzik O, et al. Fully Automated, Semantic Segmentation of Whole-Body (18)F-FDG PET/CT Images Based on Data-Centric Artificial Intelligence. J Nucl Med 2022;63:1941-1948.
3. Isensee F, Jaeger PF, Kohl SAA, et al. nnU-Net: a self-configuring method for deep learning-based biomedical image segmentation. Nat Methods 2021;18:203-211.
4. Kesner AL, Carter LM, Ramos JCO, et al. MIRD Pamphlet No. 28, Part 1: MIRDcalc-A Software Tool for Medical Internal Radiation Dosimetry. J Nucl Med 2023;64:1117-1124.
5. Carter LM, Ocampo Ramos JC, Olguin EA, et al. MIRD Pamphlet No. 28, Part 2: Comparative Evaluation of MIRDcalc Dosimetry Software Across a Compendium of Diagnostic Radiopharmaceuticals. J Nucl Med 2023;64:1295-1303.
6. Kesner AL, Carter LM, Bolch WE. Addendum to MIRD Pamphlet No. 28. J Nucl Med 2023;64:1668.
7. Icrp. Radiation dose to patients from radiopharmaceuticals. Addendum 3 to ICRP Publication 53. ICRP Publication 106. Approved by the Commission in October 2007. Ann ICRP 2008;38:1-197.
8. Wasserthal J, Breit HC, Meyer MT, et al. TotalSegmentator: Robust Segmentation of 104 Anatomic Structures in CT Images. Radiol Artif Intell 2023;5:e230024.
