# Supplementary material for: Oral [18F]-Fluoro-Thia-Heptadecanoic Acid Positron Emission Tomography Reveals Mesenteric-to-Central Lymphatic Flow
Source: Gastro Hep Adv. 2026 Apr 8;5(6):100956. doi: 10.1016/j.gastha.2026.100956 (PMC13186019; doi:10.1016/j.gastha.2026.100956)
Supplement: Extended PDF [file mmc3.pdf]

Gastro Hep Advances 2026;5:100956

RESEARCH LETTER

Oral [^{18}F]-Fluoro-Thia-Heptadecanoic Acid Positron Emission Tomography Reveals Mesenteric-to-Central Lymphatic Flow

Interstitial fluid continuously arises in tissues and returns to plasma through the one-way flow system of the lymphatic vasculature. The body's central lymphatic vessel returns lymph to the venous circulation.[1,2] Lymphatic leaks or pathologic collaterals can arise from congenital or acquired pathologies like chylothorax or protein-losing enteropathy.[1,2] For management of these conditions, central lymphatic imaging is essential. Current state-of-the-art imaging relies on dynamic contrast-enhanced magnetic resonance lymphangiography.[1,3,4] Due to its invasive nature, this procedure is rarely employed in healthy individuals.[4] Dynamic contrast-enhanced magnetic resonance lymphangiography of abdominal organs such as the liver or the mesentery is technically challenging and limited to a few referral centers. The majority of what is known about central lymphatic outflow in healthy subjects arises from cadaveric studies.[1,5,6] Here, following informed consent in a protocol approved by Washington University's Institutional Review Board (protocol #202403135) and the Radioactive Drug Research Committee (protocol #981F), we evaluated the fatty acid radiotracer [^{18}F]-fluoro-thia-heptadecanoic acid ([^{18}F]-FTHA) as a positron emission tomography (PET) agent for imaging of the mesenteric and central lymphatic vasculature. When given orally, [^{18}F]-FTHA incorporates into chylomicrons[7,8] that enter the lymph of the small bowel and drain through the mesenteric lymphatics to the TD to deliver fatty nutrients to the body.[2]

We combined a 1.4 mCi (range, 1.2–1.5 mCi) dose of [^{18}F]-FTHA into a 236 mL liquid mixed meal based on the nutritional drink BOOST that was consumed by each participant over a 10-minute interval, followed by ingestion of 118 mL of the same drink without [^{18}F]-FTHA and 118 mL of water. Serial PET/computed tomography (CT) imaging was initiated 20 minutes after the drink was started. Radiation dosimetry and biodistribution estimates were obtained in 7 healthy individuals (3 males, 4 females) by sequential whole-body continuous bed motion PET/CT acquisition from vertex to upper thigh starting from completion of the drink and repeated at 2, 4, and 6 hours to establish tracer biodistribution. Time-integrated activity coefficients (TIACs) were calculated (Supplementary Figure D) and organ-specific activity determined (Supplementary Table). TIACs for different organs from a representative individual are shown in Supplementary Figure E–H.

Tracer activity appeared immediately in the stomach and duodenum, with low contrast activity also retained in the mouth and esophagus (Figure A). Within 20 minutes after imaging began, faint activity that increased over time was apparent in the TD and at the LVJ (Figure A, blue arrows) and in mesenteric regions between small intestinal bowel loops (Figure A, red outlines). [^{18}F]-FTHA in the TD was readily segmented from the esophagus (Figure B), including in individuals with 2 TD termini (Figure C).

Activity extraluminal to the gastrointestinal tract was surmised to correspond with [^{18}F]-FTHA secretion of chylomicrons into the mesenteric and central lymphatic outflow (TD). [^{18}F]-FTHA diluted abruptly upon entering faster flowing blood, represented by a sharp drop in PET signal at the region of the LVJ (Figure A, Supplementary Figure A). [^{18}F]-FTHA activity within the TD and mesenteric lymphatics of the small intestine appeared simultaneously (Figure A). At early time points, signal in organs like the heart and liver was low (Supplementary Figure B), but following delivery of [^{18}F]-FTHA to the systemic circulation where lipoprotein lipase releases fatty acids from chylomicrons that reach the blood,[7] [^{18}F]-FTHA activity increased in these organs while remaining lower than signal in the upper gastrointestinal and TD (Supplementary Figure C). The highest organ dose and longest TIACs were observed at the stomach wall with a sex-averaged dose of 1.36 rad/mCi (Supplementary Table). The sex-averaged effective dose (103) was 0.22 rem/mCi (59.5 μSv/MBq) or 3.0 mSv per 50 MBq (Supplementary Table).

To approximate lymph flow rate, we segmented several centimeters of the terminal end of the TD starting from where it angled away from the esophagus to the LVJ (Supplementary Figure I). The initial delivery rate of [^{18}F]-FTHA across the LVJ rose at 0.00511% $\pm$ 0.00128% ingested dose (ID)/min, reaching ~0.0929% $\pm$ 0.0253% ID at 53.6 $\pm$ 9.48 minutes before declining by ~110 minutes (Figure D). Area under the curve analysis indicated that 13.7 $\pm$ 3.4% ID passed through the LVJ during the 220-minute period investigated. Distinct paths of PET signal were observed between the intestine and the TD on PET/CT, representing mesenteric lymphatic vessels (Figure E, white arrows and white dotted line).

Inclusive of our dosimetry analysis, we investigated [^{18}F]-FTHA outflow through central lymphatics in 10 healthy individuals (6 females, 4 males; age 28–62 years; BMI 20–32 kg/m^2) who had not been diagnosed with suspected lymphatic disorders. Three of the 10 healthy subjects had two TD termini (Figure F, green arrows), an uncommon but known variation in the central lymphatic system.[9] We also observed prominent contrast in a secondary trunk near the TD in at least half of the healthy participants (Figure F, red arrows),

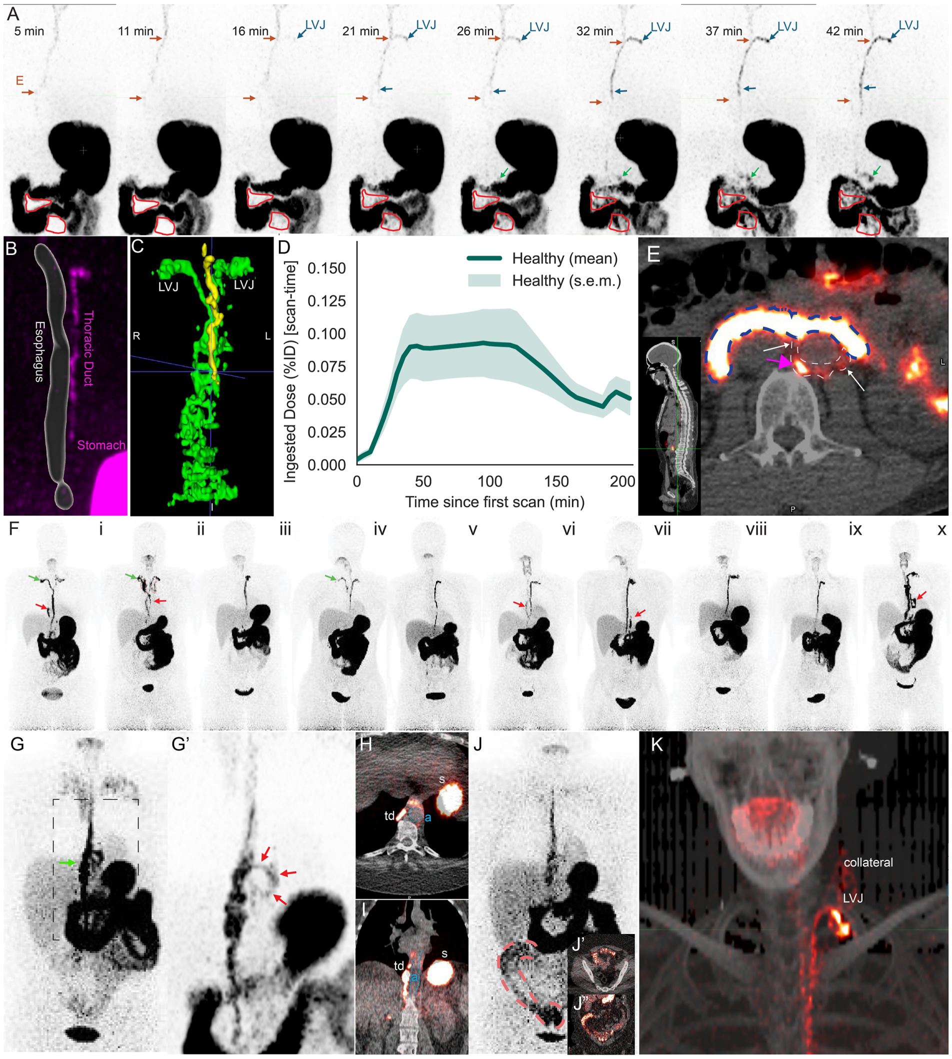

Figure. Oral [18F]-FTHA PET/CT visualizes mesenteric-to-central lymphatic transport. (A) Serial, coronal frames of maximum intensity projection (MIP) PET signal after oral [18F]-FTHA. Esophagus (E; orange arrows) and TD (blue arrows), including the LVJ of the TD near the clavicle, are annotated. Regions of mesentery between bowel loops are outlined in red. (B) TotalSegmentator-derived segmentation of esophagus (light gray) overlaid on the PET signal (magenta) demonstrates TD near esophagus. (C) Representative 3D surface rendering after voxel-wise segmentation of lymphatic tracer signal (green) vs esophageal signal (yellow) in healthy subject. (D) LVJ time–activity curve in healthy participants (percent ingested dose, %ID, over time since first scan; arithmetic mean ± SEM, $n = 10$). (E) Axial PET/CT overlay at the level of mid abdomen demonstrating the PET signal within the small intestine lumen (blue dashes), with lymphatic vessels bearing [18F]-FTHA (white arrows, white dotted lines) extending from the intestine to the TD (magenta arrow). (F) Whole-body MIPs from each healthy participant (numbered i-x) showing PET signal in the TD outflow terminating at 1 or 2 LVJs. Green arrows indicate 2 TD termini (dual LVJ) variants; red arrows indicate prominent secondary lymphatic trunk outflow alongside the TD. (G) At approximately 100 minutes, MIP PET in a participant with lifelong PLE showing an engorged TD segment (green arrow) without signal reaching a distinct LVJ near the clavicle; boxed region indicates the area magnified in (G'). (G') Magnified MIP highlighting abnormal central lymphatic anatomy along with lymphatic collateral channel (red arrows). (H and I) Axial (H) or coronal (I) PET/CT overlay in PLE patient demonstrating abnormal [18F]-FTHA-containing pathway looping around the esophagus and descending aorta (a, blue dashed); stomach (s). (J) Delayed MIP at approximately 220 minutes in the same PLE patient showing prominent colonic [18F]-FTHA tracer activity (red dashed outline), with PET/CT confirmation on (J') axial and (J") coronal views. (K) MIP PET/CT in a primary lymphedema patient demonstrating TD and LVJ signal, with collateral signal further extending aberrantly into the neck. PLE, protein-losing enteropathy.

along with additional lymphatic collaterals in some individuals (Figure F, panel x). These secondary trunks were apparent after surface rendering to remove stomach and intestinal signal (Figure C). Segmentation of the esophagus confirmed that the signal within the collateral trunk was distinct from the esophagus (Figure C). These secondary excursions are not well described in existing literature and suggest that central lymphatic pathways are not yet fully defined in healthy humans. It is possible that lymph trunks thought to feed into the TD that lack valves[10] receive reflux from the TD in some settings, such as in the postprandial state studied here.

We next recruited a patient with lifelong protein-losing enteropathy (Figure G–J). When given oral [^{18}F]-FTHA in our protocol, findings in this patient suggested possible central lymphatic flow obstruction with secondary flow via lymphatic collaterals. [^{18}F]-FTHA activity was present in a highly dilated segment of the TD (Figure G, green arrow; magnified in Figure G’) without activity apparent at the LVJ. Vague signal in the pulmonary system was present (Figure G), possibly representing retrograde flow into pulmonary lymphatics. At the site of TD dilation, activity adjacent to the esophagus and descending aorta was identified (Figure H and I, aorta outlined in blue), which could represent collateral flow. By 220 minutes, a signal was observed in the colon (Figure J and J”), an unusual outcome for dietary fatty acid in normal subjects, but observed in one of the healthy participants (Figure F, panel x). [^{18}F]-FTHA signal (dietary fat) in the colon may be consistent with lymphatic pathologies driving protein-losing enteropathy and account for the patient’s propensity to have >5 bowel movements per day.

Finally, a primary lymphedema patient transported lymph to the LVJ at the subclavian vein, but collaterals carrying [^{18}F]-FTHA extended from the LVJ into the neck (Figure K), which may reflect backflow into cervical lymphatic channels due to partial LVJ obstruction.

We conclude that oral [^{18}F]-FTHA–PET/CT facilitates safe and effective noninvasive, dynamic imaging of mesenteric-to-central lymphatic transport and has potential to illuminate a better understanding of mesenteric-to-central lymph flow in health and disease.

DANIEL D. LEE[1]
RICHARD LAFOREST[2]
ALEXANDER USHINSKY[2]
MICHAEL L. NICKELS[2]
ROBERT J. GROPLER[2]
GWENDALYN J. RANDOLPH[1]
THE FTHA STUDY WORKING GROUP
[1]Department of Pathology and Immunology, Washington University School of Medicine, St. Louis, Missouri
[2]Department of Radiology, Mallinckrodt Institute of Radiology, Washington University School of Medicine, St. Louis, Missouri

Correspondence:
Address correspondence to: Gwendalyn J. Randolph, PhD, Washington University School of Medicine, 425 S. Euclid Avenue, Campus Box 8118 86 10, St. Louis, Missouri 63110. e-mail: gjrandolph@wustl.edu.

Supplementary Materials

Material associated with this article can be found, in the online version, at https://doi:10.1016/j.gastha.2026.100956.

References

1. Mehrara BJ, et al. J Clin Invest 2023;133:e171582.
2. Tso P, et al. Nat Rev Gastroenterol Hepatol 2025;22:127–145.
3. Zheng Q, et al. Radiology 2020; 296:202–207.
4. Garlisi Torales LD, et al. J Clin Invest 2024;134:e172839.
5. Davis HK. Am J Anat 1915; 17:211–244.
6. Zorzetto NL, et al. J Morphol 1977; 153:363–369.
7. Labbe SM, et al. Am J Physiol Endocrinol Metab 2011; 300:E445–E453.
8. Carpentier AC. Physiology (Bethesda) 2024;39:0.
9. Ratnayake CBB, et al. J Anat 2018; 233:1–14.
10. Langford RJ. J Craniomaxillofac Surg 2002;30:121–124.

Abbreviations used in this paper: **[^{18}F]-FTHA, [^{18}F]-fluoro-thia-heptadecanoic acid; CT, computed tomography; ID, ingested dose; LVJ, lymphovenous junction; PET, positron emission tomography; TD, thoracic duct; TIACs, time-integrated activity coefficients**

Most current article

© 2026 The Author(s). Published by Elsevier Inc. on behalf of American Gastroenterological Association Institute. This is an open access article under the CC BY license (http://creativecommons.org/licenses/by/4.0/).
2772-5723
https://doi.org/10.1016/j.gastha.2026.100956

Received February 27, 2026. Accepted April 1, 2026.

Acknowledgments:
The FTHA Study Working Group includes Heyun Lee (Department of Pathology & Immunology, Washington University, St. Louis, MO), Kitty Harrison (Mallinckrodt Institute of Radiology, Washington University, St. Louis, MO), Nicholas Dunn (Mallinckrodt Institute of Radiology, Washington University, St. Louis, MO), Christopher G. Huckstep (Department of Pathology & Immunology, Washington University, St. Louis, MO), Quazim Alayo (Department of Pathology & Immunology, Washington University, St. Louis, MO), Bernd H. Zinseleyer (Department of Pathology & Immunology, Washington University, St. Louis, MO), Shelei Pan (Department of Neurosurgery, Washington University, St. Louis, MO), Adam Neff (MIM Software Inc, Cleveland, OH), and Ben Stawicki (MIM Software Inc, Cleveland, OH). We extend gratitude to André Carpentier (Université de Sherbrooke, Quebec, Canada) and Olof Solin (University of Turku, Finland) for helpful advice and Justin Berger (Washington University) for discussion.

Authors’ Contributions:
Daniel D. Lee: Investigation, visualization: lead, data curation, formal analysis, validation, writing–first draft: equal; methodology: equal. Richard Laforest: Data curation, formal analysis, methodology, supervision, writing–first draft: equal, conceptualization, investigation, project administration, visualization, funding acquisition: supporting. Alexander Ushinsky: Resources: supporting, writing–review and editing: lead. Michael L. Nickels: Methodology: equal, writing–review and editing: supporting. Robert J. Gropler: Conceptualization, project administration, supervision, writing–review and editing, methodology: equal, resources: lead, funding acquisition, investigation: supporting. Gwendalyn J. Randolph: Funding acquisition: lead, conceptualization, project administration, supervision, writing–first draft: equal, investigation, methodology, visualization: supporting.

Conflicts of Interest:
The authors disclose no conflicts.

Funding:
This work was funded by National Institutes of Health (NIH) grant DP1DK130660 to Gwendalyn J.

Randolph. Daniel D. Lee was supported in part by NIH grant T32 HL007081. Additional funding for resources at Washington University included the PET Radiotracer Translation and Resource Center (P41EB025815), Digestive Diseases Research Core Center (P30DK052574), and Washington University Nutrition Obesity Research Center (P30DK056341).

Ethical Statement:
Studies were approved by the Washington University's Institutional Review Board (protocol #202403135) and the Radioactive Drug Research Committee (protocol #981F).

Data Transparency Statement:
Data and supporting materials will be made available to other researchers upon reasonable request to the corresponding author.

Reporting Guidelines:
European Association of Nuclear Medicine guidance standards for clinical dosimetry reporting were followed.

Gastro Hep Advances, Volume 5

Supplemental information

Oral [^{18}F]-Fluoro-Thia-Heptadecanoic Acid Positron Emission Tomography Reveals Mesenteric-to-Central Lymphatic Flow

Daniel D. Lee, Richard Laforest, Alexander Ushinsky, Michael L. Nickels, Robert J. Gropler, and Gwendalyn J. Randolphthe FTHA Study Working Group

Supplemental Text - Methods

Participant characteristics and recruitment

This observational study recruited study participants ages 21–80. Approval was granted by Washington University institutional review board, protocol #202403135 and the Radioactive Drug Research Committee, protocol #981F. Written informed consent was obtained prior to enrollment. Healthy-subject inclusion criteria required no known history of, or clinical suspicion for, cardiac or lymphatic vascular disease. A second cohort included participants with lymphatic or related congenital anomalies. Exclusion criteria included pregnancy, body weight >300 lb, recreational drug use, current participation in other investigational or radiopharmaceutical studies, and inability to tolerate PET/CT (e.g., claustrophobia or inability to remain supine). Medications and supplements were reviewed and eligibility was determined case-by-case. Participants completed a brief health survey and focused physical examination prior to imaging. Twelve adult participants (10 healthy, 2 in lymphatic disorders cohort) were enrolled (8 females, 4 males). Adverse event monitoring included real-time symptom reporting, baseline and end-of-scan vital signs, structured symptom checklists, and pre-/post-scan electrocardiograms. Nausea, reflux, emesis, abdominal discomfort, and allergic symptoms were specifically queried. No adverse events were encountered.

Synthesis of FTHA

14-(R,S)-[^{18}F] Fluoro-6-thia-heptadecanoic acid (FTHA) was prepared at the Washington University Cyclotron Facility and Nuclear Pharmacy using a modification of a published method[1]. Briefly, Kryptofix-222 assisted radiofluorination of the tosylate precursor was followed by hydrolysis with KOH. Radiosynthesis was performed on a GE FX-2N module, and purified by semi-preparative HPLC and sterile filtered to provide a ready to use final product (total synthesis time ~80 minutes).

Imaging protocol and tracer administration

A 16-oz formulated liquid mixed meal validated in prior chylomicron tracer studies was prepared in the Washington University Clinical Research and Translational Unit metabolic kitchen (10.8 g Sol Carb, 413 g chocolate BOOST® Plus, 2.6 g canola oil, 0.116 g lecithin, 24 g water). In the Mallinckrodt Institute of Radiology Center for Clinical Imaging and Research (CCIR), approximately 1.4 mCi (range 1.2–1.5 mCi) of [^{18}F]-FTHA was added to an 8-oz portion in a sealed cup with radiation shielding; overnight-fasted participants consumed the tracer-containing portion over 10 minutes after an overnight fast, followed by 4 oz of the drink without tracer and then 4 oz of water. PET/CT was performed on a Siemens Biograph Vision-600. Imaging began ~10 minutes after completion of the drink (~20 minutes after drink initiation). The first hour was acquired as serial whole-body continuous-bed-motion (CBM) sweeps with frame durations of approximately 5–10 minutes per sweep (5–10 sweeps total, depending on protocol). Additional whole-body CBM acquisitions were obtained at ~2 hours (15 minutes) and at later timepoints (e.g., 4 hours and 6 hours; 30 minutes each). Participants exited the scanner between sessions and were repositioned on return. For anatomic localization and attenuation correction, a low-dose CT was acquired at each imaging session (120 kVp, 38.4 mm total collimation, 17 mAs effective; CT reconstructed at 3-mm slice thickness per protocol).

Imaging analysis

The primary high-activity compartments prioritized for dosimetry quantification were stomach contents, small intestine contents, and liver; kidneys, spleen, urinary bladder, and heart were also evaluated. Liver, kidneys, spleen, and stomach were segmented using MOOSE3 AI software[2, 3]. Masses of liver, kidneys, and spleen, were estimated from segmented volumes assuming a tissue density of 1.05 g/cm³. A single volume of interest (VOI) encompassing the whole heart was used to sample cardiac activity (myocardium and blood pool were not separable on visual assessment). Stomach, urinary bladder, and small intestine activity were quantified as total activity within PET-derived VOI volumes.

Organ time-integrated activities (TIAs, formerly known as residence times, in hours) for each organ were computed from time–activity data expressed as percent injected dose per organ using trapezoidal numerical integration and fit with exponential functions to extrapolate beyond the last imaging time point. For stomach contents, the time–activity curve was additionally fitted to a bi-exponential model using Microsoft Excel Solver and analytically integrated to obtain the TIA. The calculated TIAs were entered into MIRDCalc (v1)[4-6] for F-18 and using the either the adult ICRP human male and female anthropomorphic models, as appropriate. For cardiac portioning, a fraction of heart wall residence time was assigned to the heart content as defined by the ratio of heart content to heart wall mass, where the heart content and mass were taken from ICRP-106[7]. All unaccounted activity in the organs was assigned to the reminder of the body. Organ radiation dose and Effective Dose (IRCP-103) were reported.

Esophagus and gastrointestinal tract were segmented using MOOSE3 AI software[2] or TotalSegmentator[8], while voxel-by-voxel segmentation and surface rendering was performed on ITK-Snap. For 3D visualization, segmentation label maps (NIfTI) and the corresponding CT and PET image volumes (converted from DICOM to image stacks) were imported into Imaris (v10.1.1) using the Imaris File Converter and rendered as overlaid volumetric channels to display anatomical context and segmented structures in three dimensions.

Image registration and calculations

Serial PET datasets (dynamic first-hour frames and subsequent CBM acquisitions) were aligned across timepoints to correct for inter-scan repositioning using CT for anatomical guidance (MIM v7.3.7). The first-hour plus ~2-hour aligned PET/CT dataset was used as the reference, and later frames were registered using rigid followed by deformable registration; transforms were applied to the PET data to enable time-consistent sampling. A 3D VOI encompassing the thoracic duct from the diaphragmatic hiatus to the LVJ was delineated on a timepoint with clear duct activity and deformably propagated to all frames. Propagated VOIs were reviewed and edited frame-by-frame to exclude adjacent non-duct activity and to confirm anatomical concordance on PET/CT overlay.

VOI activity at each timepoint was obtained from the VOI export (using mean activity concentration and VOI volume). Time was expressed as minutes from the first scan. Exported PET values were decay-corrected to administration and converted to scan-time activity using the physical decay of F-18 ($T_{½}$ = 109.77 min). Percent injected dose was calculated as $\%ID_{\text{scan}} = 100 \times A_{\text{VOI,scan}}/A_{\text{inj}}$, with A_{inj} converted to Bq. When bilateral LVJ VOIs were available, LVJ_{Total} was computed per timepoint by summing left and right VOI activities (and volumes), and %ID were calculated from the combined VOI. For healthy-group summaries, individual %ID

time–activity curves were restricted to 0–240 min and linearly interpolated onto a uniform 5-min grid; interpolation was limited to each subject's observed time range (no extrapolation). At each grid timepoint, the group mean along with standard error of mean was calculated across available subjects. Because LVJ time–activity curves frequently exhibited a sustained plateau rather than a sharp peak, a "near-max time" was defined as the first grid time at which the group-mean curve reached ≥95% of its maximum value and remained above this threshold for ≥3 consecutive grid points (15 minutes). Group peak %ID was defined as the maximum of the group-mean curve. Delivery rate was defined as the time-derivative of %ID (%ID/min) using a rolling k-point linear regression (k=3) by fitting $\%ID = mt + b$ within each window and taking the maximum positive slope m as the peak rolling delivery rate, reported at the window's median time. Peak delivery time was estimated at the window's maximum.

References

1. Savisto N, Viljanen T, Kokkomaki E, et al. Automated production of [(18) F]FTHA according to GMP. J Labelled Comp Radiopharm 2018;61:84-93.
2. Shiyam Sundar LK, Yu J, Muzik O, et al. Fully Automated, Semantic Segmentation of Whole-Body (18)F-FDG PET/CT Images Based on Data-Centric Artificial Intelligence. J Nucl Med 2022;63:1941-1948.
3. Isensee F, Jaeger PF, Kohl SAA, et al. nnU-Net: a self-configuring method for deep learning-based biomedical image segmentation. Nat Methods 2021;18:203-211.
4. Kesner AL, Carter LM, Ramos JCO, et al. MIRD Pamphlet No. 28, Part 1: MIRDcalc-A Software Tool for Medical Internal Radiation Dosimetry. J Nucl Med 2023;64:1117-1124.
5. Carter LM, Ocampo Ramos JC, Olguin EA, et al. MIRD Pamphlet No. 28, Part 2: Comparative Evaluation of MIRDcalc Dosimetry Software Across a Compendium of Diagnostic Radiopharmaceuticals. J Nucl Med 2023;64:1295-1303.
6. Kesner AL, Carter LM, Bolch WE. Addendum to MIRD Pamphlet No. 28. J Nucl Med 2023;64:1668.
7. Icrp. Radiation dose to patients from radiopharmaceuticals. Addendum 3 to ICRP Publication 53. ICRP Publication 106. Approved by the Commission in October 2007. Ann ICRP 2008;38:1-197.
8. Wasserthal J, Breit HC, Meyer MT, et al. TotalSegmentator: Robust Segmentation of 104 Anatomic Structures in CT Images. Radiol Artif Intell 2023;5:e230024.

Table S1. Human radiation dose for 18F-FTHA in humans. Doses can be converted to SI units by dividing by 3.7.

Organ dose (rad/mCi)	1-F	3-F	4-F	010-F	Female		2-M	5-M	6-M	Male		Sex Averaged	
Organ					Average +/- SD					Average +/- SD		Average +/- SD	
Adipose tissue	0.034	0.038	0.033	0.029	0.033	0.004	0.037	0.036	0.036	0.036	0.001	0.033	0.003
Adrenals	0.145	0.122	0.152	0.171	0.147	0.020	0.101	0.105	0.111	0.106	0.005	0.147	0.027
Bone - endosteal cells	0.033	0.038	0.031	0.028	0.032	0.004	0.026	0.025	0.023	0.025	0.002	0.032	0.005
Bone red marrow	0.053	0.056	0.052	0.051	0.053	0.002	0.043	0.042	0.041	0.042	0.001	0.053	0.006
Brain	0.020	0.027	0.018	0.012	0.019	0.006	0.016	0.015	0.012	0.014	0.002	0.019	0.005
Breast tissue	0.040	0.040	0.039	0.040	0.040	0.001	0.000	0.000	0.000	0.000	0.000	0.040	0.021
Colon - left	0.067	0.069	0.068	0.068	0.068	0.001	0.149	0.154	0.163	0.155	0.007	0.068	0.047
Colon - rectosigmoid	0.055	0.063	0.058	0.051	0.057	0.005	0.035	0.033	0.034	0.034	0.001	0.057	0.013
Colon - right	0.054	0.058	0.054	0.053	0.055	0.002	0.065	0.067	0.067	0.066	0.001	0.055	0.006
Esophagus	0.069	0.062	0.067	0.074	0.068	0.005	0.065	0.067	0.070	0.067	0.002	0.068	0.004
Eye lens	0.016	0.021	0.014	0.010	0.015	0.004	0.012	0.011	0.009	0.011	0.002	0.015	0.004
Gallbladder wall	0.129	0.114	0.138	0.154	0.134	0.017	0.088	0.094	0.093	0.092	0.003	0.134	0.025
Heart wall	0.128	0.085	0.105	0.165	0.121	0.034	0.107	0.118	0.135	0.120	0.014	0.121	0.025
Kidneys	0.139	0.122	0.147	0.170	0.145	0.020	0.095	0.097	0.115	0.103	0.011	0.145	0.027
Liver	0.131	0.114	0.144	0.158	0.137	0.019	0.093	0.116	0.117	0.109	0.013	0.137	0.022
Lung - ICRP133	0.057	0.054	0.055	0.059	0.056	0.002	0.050	0.051	0.051	0.051	0.001	0.056	0.003
Muscle	0.033	0.039	0.032	0.028	0.033	0.004	0.026	0.025	0.023	0.025	0.001	0.033	0.005
Oral mucosa	0.031	0.041	0.027	0.020	0.030	0.009	0.025	0.023	0.019	0.022	0.003	0.030	0.007
Ovaries	0.056	0.065	0.059	0.051	0.058	0.006	0.000	0.000	0.000	0.000	0.000	0.058	0.031
Pancreas	0.223	0.184	0.234	0.264	0.226	0.033	0.185	0.193	0.202	0.194	0.009	0.226	0.030
Pituitary gland	0.022	0.030	0.020	0.014	0.022	0.007	0.017	0.016	0.013	0.015	0.002	0.022	0.006
Prostate	0.000	0.000	0.000	0.000	0.000	0.000	0.036	0.032	0.034	0.034	0.002	0.000	0.018
Salivary glands	0.022	0.029	0.020	0.014	0.021	0.006	0.017	0.015	0.013	0.015	0.002	0.021	0.006
Skin	0.024	0.028	0.023	0.020	0.024	0.003	0.019	0.019	0.017	0.018	0.001	0.024	0.004
Small intestine	0.192	0.184	0.202	0.221	0.200	0.016	0.196	0.206	0.214	0.205	0.009	0.200	0.013
Spleen	0.234	0.202	0.242	0.269	0.237	0.028	0.124	0.168	0.164	0.152	0.024	0.237	0.051
Stomach Wall	1.388	0.936	1.465	1.717	1.376	0.325	1.262	1.302	1.436	1.333	0.091	1.376	0.237
Testes	0.000	0.000	0.000	0.000	0.000	0.000	0.019	0.017	0.014	0.017	0.002	0.000	0.009
Thymus	0.035	0.040	0.031	0.030	0.034	0.004	0.029	0.029	0.027	0.028	0.001	0.034	0.004
Thyroid	0.027	0.034	0.025	0.020	0.027	0.006	0.023	0.022	0.020	0.022	0.002	0.027	0.005
Urinary bladder wall	0.061	0.068	0.080	0.055	0.066	0.011	0.060	0.052	0.065	0.059	0.007	0.066	0.009
Uterus	0.062	0.070	0.068	0.057	0.064	0.006	0.000	0.000	0.000	0.000	0.000	0.064	0.035
Whole body target	0.040	0.043	0.038	0.037	0.040	0.003	0.034	0.033	0.032	0.033	0.001	0.040	0.004
Effective Dose (rem/mCi)	0.221	0.168	0.232	0.283	0.226	0.048	0.202	0.208	0.225	0.212	0.012	0.220	0.035

Table S1. Human radiation dose for 18F-FTHA in humans. Doses can be converted to SI units by dividing by 3.7.

Organ dose (rad/mCi)	1-F	3-F	4-F	010-F	Female		2-M	5-M	6-M	Male		Sex Averaged	
Organ					Average +/- SD					Average +/- SD		Average +/- SD	
Adipose tissue	0.034	0.038	0.033	0.029	0.033	0.004	0.037	0.036	0.036	0.036	0.001	0.033	0.003
Adrenals	0.145	0.122	0.152	0.171	0.147	0.020	0.101	0.105	0.111	0.106	0.005	0.147	0.027
Bone - endosteal cells	0.033	0.038	0.031	0.028	0.032	0.004	0.026	0.025	0.023	0.025	0.002	0.032	0.005
Bone red marrow	0.053	0.056	0.052	0.051	0.053	0.002	0.043	0.042	0.041	0.042	0.001	0.053	0.006
Brain	0.020	0.027	0.018	0.012	0.019	0.006	0.016	0.015	0.012	0.014	0.002	0.019	0.005
Breast tissue	0.040	0.040	0.039	0.040	0.040	0.001	0.000	0.000	0.000	0.000	0.000	0.040	0.021
Colon - left	0.067	0.069	0.068	0.068	0.068	0.001	0.149	0.154	0.163	0.155	0.007	0.068	0.047
Colon - rectosigmoid	0.055	0.063	0.058	0.051	0.057	0.005	0.035	0.033	0.034	0.034	0.001	0.057	0.013
Colon - right	0.054	0.058	0.054	0.053	0.055	0.002	0.065	0.067	0.067	0.066	0.001	0.055	0.006
Esophagus	0.069	0.062	0.067	0.074	0.068	0.005	0.065	0.067	0.070	0.067	0.002	0.068	0.004
Eye lens	0.016	0.021	0.014	0.010	0.015	0.004	0.012	0.011	0.009	0.011	0.002	0.015	0.004
Gallbladder wall	0.129	0.114	0.138	0.154	0.134	0.017	0.088	0.094	0.093	0.092	0.003	0.134	0.025
Heart wall	0.128	0.085	0.105	0.165	0.121	0.034	0.107	0.118	0.135	0.120	0.014	0.121	0.025
Kidneys	0.139	0.122	0.147	0.170	0.145	0.020	0.095	0.097	0.115	0.103	0.011	0.145	0.027
Liver	0.131	0.114	0.144	0.158	0.137	0.019	0.093	0.116	0.117	0.109	0.013	0.137	0.022
Lung - ICRP133	0.057	0.054	0.055	0.059	0.056	0.002	0.050	0.051	0.051	0.051	0.001	0.056	0.003
Muscle	0.033	0.039	0.032	0.028	0.033	0.004	0.026	0.025	0.023	0.025	0.001	0.033	0.005
Oral mucosa	0.031	0.041	0.027	0.020	0.030	0.009	0.025	0.023	0.019	0.022	0.003	0.030	0.007
Ovaries	0.056	0.065	0.059	0.051	0.058	0.006	0.000	0.000	0.000	0.000	0.000	0.058	0.031
Pancreas	0.223	0.184	0.234	0.264	0.226	0.033	0.185	0.193	0.202	0.194	0.009	0.226	0.030
Pituitary gland	0.022	0.030	0.020	0.014	0.022	0.007	0.017	0.016	0.013	0.015	0.002	0.022	0.006
Prostate	0.000	0.000	0.000	0.000	0.000	0.000	0.036	0.032	0.034	0.034	0.002	0.000	0.018
Salivary glands	0.022	0.029	0.020	0.014	0.021	0.006	0.017	0.015	0.013	0.015	0.002	0.021	0.006
Skin	0.024	0.028	0.023	0.020	0.024	0.003	0.019	0.019	0.017	0.018	0.001	0.024	0.004
Small intestine	0.192	0.184	0.202	0.221	0.200	0.016	0.196	0.206	0.214	0.205	0.009	0.200	0.013
Spleen	0.234	0.202	0.242	0.269	0.237	0.028	0.124	0.168	0.164	0.152	0.024	0.237	0.051
Stomach Wall	1.388	0.936	1.465	1.717	1.376	0.325	1.262	1.302	1.436	1.333	0.091	1.376	0.237
Testes	0.000	0.000	0.000	0.000	0.000	0.000	0.019	0.017	0.014	0.017	0.002	0.000	0.009
Thymus	0.035	0.040	0.031	0.030	0.034	0.004	0.029	0.029	0.027	0.028	0.001	0.034	0.004
Thyroid	0.027	0.034	0.025	0.020	0.027	0.006	0.023	0.022	0.020	0.022	0.002	0.027	0.005
Urinary bladder wall	0.061	0.068	0.080	0.055	0.066	0.011	0.060	0.052	0.065	0.059	0.007	0.066	0.009
Uterus	0.062	0.070	0.068	0.057	0.064	0.006	0.000	0.000	0.000	0.000	0.000	0.064	0.035
Whole body target	0.040	0.043	0.038	0.037	0.040	0.003	0.034	0.033	0.032	0.033	0.001	0.040	0.004
Effective Dose (rem/mCi)	0.221	0.168	0.232	0.283	0.226	0.048	0.202	0.208	0.225	0.212	0.012	0.220	0.035
